# Dietary Advanced Glycation End Products: Digestion, Metabolism and Modulation of Gut Microbial Ecology

**DOI:** 10.3390/nu11020215

**Published:** 2019-01-22

**Authors:** Matthew Snelson, Melinda T. Coughlan

**Affiliations:** Department of Diabetes, Central Clinical School, Monash University, Alfred Medical Research and Education Precinct, 3004 Melbourne, Australia; melinda.coughlan@monash.edu

**Keywords:** advanced glycation end products, maillard reaction products, heat-treated diets, microbiota, short-chain fatty acids

## Abstract

The formation of advanced glycation end products (AGEs) in foods is accelerated with heat treatment, particularly within foods that are cooked at high temperatures for long periods of time using dry heat. The modern processed diet is replete with AGEs, and excessive AGE consumption is thought to be associated with a number of negative health effects. Many dietary AGEs have high molecular weight and are not absorbed in the intestine, and instead pass through to the colon, where they are available for metabolism by the colonic bacteria. Recent studies have been conducted to explore the effects of AGEs on the composition of the gut microbiota as well as the production of beneficial microbial metabolites, in particular, short-chain fatty acids. However, there is conflicting evidence regarding the impact of dietary AGEs on gut microbiota reshaping, which may be due, in part, to the formation of alternate compounds during the thermal treatment of foods. This review summarises the current evidence regarding dietary sources of AGEs, their gastrointestinal absorption and role in gut microbiota reshaping, provides a brief overview of the health implications of dietary AGEs and highlights knowledge gaps and avenues for future study.

## 1. Introduction

**Figure 1:**
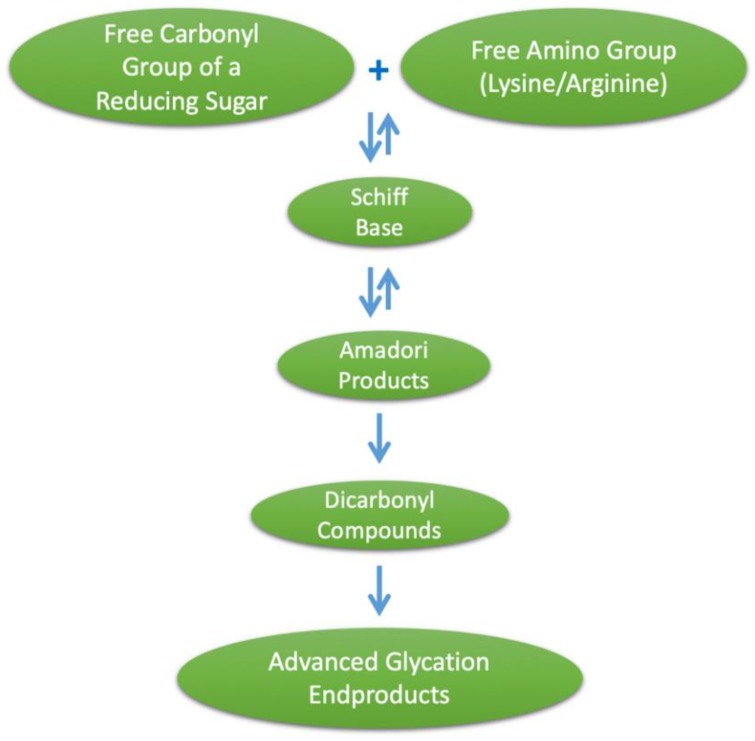
Formation of advanced glycation end products (AGEs).

Advanced glycation end products (AGEs) is a term used to describe a heterogeneous group of compounds that are formed through a series of nonenzymatic reactions, together termed the Maillard reaction, involving an amino acid with a free amino group (such as lysine or arginine) and a free carbonyl group of a reducing sugar. This initial amine–carbonyl reaction results in an imine, often referred to as a Schiff base, which undergoes rearrangement to form an intermediate glycation product, the Amadori product, which is a more stable α-ketoamine. These Schiff bases and Amadori products are initial glycation products, and at this stage are reversible. Amadori products can also undergo degradation to form dicarbonyl compounds, such as methylglyoxal (MG), and then undergo further degradation, oxidation, reduction and condensation reactions, leading to irreversible AGE formation [1], as illustrated in Figure 1. Major AGEs present in vivo or from exogenous sources include methylglyoxal–hydroimidazalone (MG-H1), carboxymethyl–lysine (CML), carboxyethyl–lysine (CEL), pentosidine and pyrraline [2]. Given the heterogeneity of AGEs formed, it is often difficult to fully quantify their formation, and subsequently CML, CEL and pentosidine are commonly used as biomarkers for the glycation process [3]. Furthermore, the heterogeneity of AGEs formed during the heat treatment of foods is greater than that of those formed physiologically, and the diversity of dietary AGEs has hindered efforts to study their effects [4]. It should be noted that the AGE pyrraline is formed exclusively within food products and thus measuring pyrraline may provide a better indicator of the effects of AGEs of exclusively dietary origin [5]. The objective of this review is to describe the food sources, absorption and health effects of dietary AGEs and provide a summary of in vitro and in vivo studies that have provided high AGE intervention and assessed the composition of the gut microbiome.

## 2. Endogenous Sources of AGEs

AGE-modified proteins accumulate within the body with aging and are thought to play a role in a number of age-related diseases including diabetes and neurodegenerative and cardiovascular diseases [6]. Endogenous AGE formation is accelerated during circumstances of high plasma glucose, such as is prevalent in diabetes [7]. Furthermore, there is evidence to indicate that excessive dietary intake of sugars, particularly fructose, contributes to endogenous AGE formation [8]. In situations of oxidative stress, oxidation of fatty acids and glucose can lead to the production of reactive carbonyls, such as MG, which contribute to AGE formation [9].

## 3. Exogenous Sources of AGEs

In addition to the endogenous formation of AGEs that can occur as described above, exogenous ingestion of AGEs can significantly contribute to their accumulation. Cigarette smoke contains glycation products that are highly reactive and lead to the formation of AGEs [10], which may contribute to the increased AGE accumulation in serum [11] and tissue [12] observed in cigarette smokers. AGEs are also formed in foods, and their formation is highly dependent on cooking methods. The heat treatment of food results in the generation of Maillard reaction products (MRPs), including AGEs, which improve the aroma and flavour of food products [2]. Food processing and cooking techniques that utilise dry heat (frying, roasting, baking, grilling, barbecuing) result in greater AGE formation compared with techniques that use lower temperatures for longer periods of time with higher water content, such as boiling or steaming [13,14]. Bread is a well-known source of MRPs, including AGEs, and reducing baking temperature from 250 °C to 200 °C resulted in a 20% lower accumulation of CML [15]. Higher pH levels can also increase the formation of AGEs, as the alkaline conditions promote amino groups being in the basic deprotonated form, increasing reactivity [16]. Pretreating foods prior to cooking with acidic solutions, such as marinating with vinegar or lemon, to lower their pH has been demonstrated to reduce the formation of AGEs in food products [17].

The modern Western diet is replete with heat-treated foods that contribute to the intake of AGEs [18]. Foods that contribute large quantities of AGEs to the modern diet include processed cereal products such as biscuits, bakery products and extruded breakfast cereals [19]; powdered milk; and fish, chicken or meat cooked with dry heat [13]. It is estimated that modern dietary intake ranges from 25 to 75 mg/d for AGEs and 500 to 1200 mg/d for Amadori products, with milk and bakery products being the major contributors [20]. The highest concentrations of CML, CEL and MG-H1 were found in biscuits, cereal products, high-heat-processed meats, peanuts and peanut butter [13]. Dietary AGE intake has been shown to correlate with plasma-free AGEs, but not protein-bound AGEs in the plasma [21], suggesting that plasma protein-bound AGEs are more indicative of endogenous AGE formation. While exogenous and endogenous AGEs are considered to be two separate sources, recently the observation has been made that they may act synergistically and contribute to an overall glycotoxin burden, which may increase the detrimental health effects of AGEs [18].

## 4. Exogenous AGEs and Health

Numerous studies support the idea that dietary consumption of AGEs contributes to oxidative stress and inflammation in animal models; however, results have been less consistent in human trials [22]. In humans, Vlassara et al. observed that two weeks of a high-AGE diet was associated with increases in circulating C-reactive protein (CRP) and tumour necrosis factor alpha (TNF-α) protein and expression in peripheral blood mononuclear cells [23], whilst Semba et al. found no effect of six weeks of high-AGE feeding on CRP, interleukin 6 (IL-6) or TNF-α receptors [24]. Two-week consumption of a diet high in AGEs was associated with an increase in inflammatory markers and albuminuria in a cohort of overweight and obese but otherwise healthy people [25]. A recent meta-analysis of randomised controlled trials utilising a low-AGE diet was associated with significant reductions in TNF-α and 8-isoprostane levels [26]. It is interesting to note that even a single high-AGE meal can result in acute endothelial dysfunction [27].

Excessive AGE consumption in mice has been implicated in the development of hepatic inflammation in the absence of steatosis [28], and in a rat model of nonalcoholic fatty liver disease, high dietary AGEs exacerbated liver injury, inflammation and liver fibrosis [29]. Chronic AGE intake has been postulated to lead to cognitive decline and Alzheimer’s disease [30]. Several studies in animal models using both healthy animals and a 5/6 nephrectomy model of chronic kidney disease (CKD) illustrated that six weeks of high-AGE feeding increased proteinuria [31,32]. In a diabetic mouse model with db/db mice, four months of high-AGE feeding was associated with an increase in albuminuria [33]. Low-AGE diets have been shown to improve markers of inflammation and oxidative stress in haemodialysis patients [34] and those with stage 3 CKD [35]; however, neither of these studies reported on kidney function. There is mounting evidence that excessive consumption of dietary AGEs contributes to inflammation and oxidative stress, which has implications for a number of chronic disease states.

## 5. Mechanisms of AGE-Induced Damage Exogenous AGEs and Health

AGEs can exert deleterious health effects and contribute to the progression of disease states through a number of mechanisms. Crosslinking of extracellular matrix proteins by AGEs contributes to thickening of the basement membrane and glomerulosclerosis within the kidney as well as arterial stiffness, which contributes to atherosclerosis [36]. AGEs have also been observed in vitro to induce basement membrane hypertrophy of the blood–brain barrier [37], and AGE modification of matrix proteins affected cellular migration and adhesion properties of T cells in vitro [38]. Guanine and adenine nucleotides contain amino groups that are susceptible to glycation, and AGE modification of DNA can lead to single strand breaks and mutations [39], which have been suggested as potential contributors to the development of renal disease [40]. There is also evidence that AGEs can act as catalytic sites for free radical formation, contributing to further damage [41].

AGEs interact with a number of cell surface receptors, of which the receptor for AGEs (RAGE) has been the most widely studied [42]. RAGE is expressed by numerous cells, including neurons, immune cells, endothelial cells and myocytes [43]. AGE–RAGE binding leads to activation of intracellular nuclear factor of κ light-chain-enhancer in activated B cells and subsequently increases the expression of a number of proinflammatory cytokines, including TNF-α, IL-6 and interleukin 1 [44]. The ligation of RAGE by AGEs also increases nicotinamide adenine dinucleotide phosphate oxidase and the production of mitochondrial reactive oxygen species [45]. AGE ligation with RAGE is implicated in the progression of a number of disease states, including cancer, atherosclerosis, stroke, metabolic syndrome and kidney disease [46,47]. There are a number of mechanisms by which AGEs, of either endogenous or exogenous origin, may impart deleterious effects.

## 6. AGE Absorption

Early studies indicated that upon oral administration, only 10–30% of AGEs are absorbed into the systemic circulation [48,49]. AGEs can exist in either free form as a single amino acid or a free low-molecular-weight (LMW, <5 kDa) peptide, or bound to proteins, forming high-molecular-weight (HMW) compounds [18]. Free CML can be absorbed by simple diffusion [50], whilst intestinal absorption of pyrraline is considered to happen mostly as a dipeptide rather than a free amino acid [51], with the dipeptide form of pyrraline absorbed across the intestinal epithelium using peptide transporter 1 (PEPT1) [52]. Further research has identified that PEPT1 is also utilised for absorption of the dipeptide versions of CML, CEL and MG-H1 [53]. Absorption of the AGE pentosidine was greater when it was provided in its free form (in brewed coffee) than protein-bound (in bakery products) [54], and diets containing protein-bound CML were associated with significantly higher faecal excretion of CML compared with diets containing the LMW fraction [55]. Modification of proteins with AGEs reduces the digestibility of the proteins [56], limiting their ability to undergo proteolytic digestion [18]. As HMW proteins require proteolytic digestion prior to absorption [2], the LMW fractions of AGEs are more likely to be absorbed [57], with the nonabsorbed AGEs progressing through the gastrointestinal tract. Tessier et al. used 13^C^-labelled CML incorporated into 13^C^-CML- bovine serum albumin (BSA) to identify CML of exclusively dietary origin in the diets of rats for 30 days. Organ accumulation, primarily in the kidneys, and faecal excretion of 13^C^-labelled CML was observed, indicating that intestinal CML absorption from dietary protein-bound CML does occur; however, a proportion is not absorbed, instead passing through the gastrointestinal tract [58]. There is a strong correlation between dietary intake of CML and faecal excretion [55,59,60], suggesting an increased opportunity for interaction between AGEs and the colonic microbiome. Between 20% and 50% of ingested CML appears to be excreted in the faeces [59,60], suggesting that there is a proportion of ingested AGEs that are not absorbed and not defecated and may be metabolised intraluminally by the microbiome. It is recognised that knowledge of the specific absorption mechanisms of dietary AGEs is limited, and further studies are required to elucidate them [61].

## 7. Dietary AGEs and Gut Microbial Composition

### 7.1. In Vitro Studies

In vitro work utilising human faecal samples has established that some AGEs become available to be selectively metabolised by certain members of the gut microbiota. Early work showed that AGEs reduce the growth rate of *Bacillus stearothermophilus* in an in vitro assay [62], and it has been established that some bacteria possess deglycation enzymes, which indicates that bacterial metabolism of glycated proteins is a possibility [63]. When faecal samples were taken from patients with ulcerative colitis (UC) and incubated with glycated BSA, there was an increase in the abundance of sulphate-reducing bacteria (SRB) and a decrease in *Bifidobacteria* and *Eubacterium rectale* compared with faeces that were incubated with nonglycated BSA [64]. A reduction in jejunal alanine absorption has been observed in animal models of experimental colitis [65,66]; however, it is unknown whether UC affects the gastrointestinal absorption of AGE-modified amino acids. In healthy controls, incubation with glycated BSA also increased the abundance of SRB, but there was no change in other bacterial groups, indicating that the initial composition of the microbiota has an impact on how it interacts with glycated proteins [64]. Twenty-four-hour incubation of a faecal sample from one healthy human volunteer with glycated pea protein was associated with an increased abundance of *Bifidobacteria* and *Lactobacilli*, as assessed by fluorescent in situ hybridization (FISH) with genus-specific 16S rRNA-targeted oligonucleotide probes, when compared with nonglycated pea protein [67]. A similar study, which utilised the same FISH probes, found that glycated gluten was associated with an expansion of *Bifidobacteria* but a contraction of *Lactobacilli* compared with heated nonglycated gluten [68]. Consistent with the bifidogenic effect that was observed with glycated gluten and pea protein, a study that incubated faecal samples from healthy volunteers with HMW bread crust fraction showed increased *Bifidobacteria*, as assessed by plating on Beerens agar, a selective growth medium for *Bifidobacteria* [69]. A recent study analysing the effect of bread crust on the microbiome observed that there were large interindividual differences with regard to *Bifidobacteria*, but noted that there was a consistent reduction in *Enterobacteria* across subjects [70]. At the phylum level, fermentation of glycated fish protein was associated with an expansion of Firmicutes and a contraction of Bacteroidetes [71]. A study using isolated AGEs identified that CML and fructoselysine are metabolised by human colonic microbiota, whilst pyrraline is not [72]. These findings indicate that whilst glycated substrates do alter microbial composition in vitro, there is a lack of agreement between studies on the specific microbial changes, which may be due to the different glycated substrates used. A summary of in vitro fermentation studies that have shown the effects of AGEs on the human microbiome is presented in Table 1.

### 7.2. Human In Vivo Studies

Whilst in vitro work has provided some insight into the effects of AGEs on the human microbiota, there is a dearth of studies modulating the AGE content of diets in humans and assessing the composition of the microbiome in vivo. A 2014 study assessed the effects of two weeks of a diet high in AGEs on the microbiome in adolescent males using targeted qPCR to measure the changes in seven bacterial groups [73]. This study observed that a high-AGE diet was associated with a contraction in *Lactobacilli*, which was negatively correlated with CML intake, and an expansion in Enterobacteria [73]. No significant differences were observed in *Bifidobacteria*, Bacteroides, *E. rectale* or *Clostridium leptum*. More recently, a one-month low-AGE intervention relative to the patient’s regular diet, which was used as the high-AGE comparator, was undertaken in peritoneal dialysis patients [74]. This study assessed the composition of the microbiome by sequencing the V3-4 region of the 16S rRNA gene and reported that dietary AGE restriction was associated with a contraction of *Prevotella copri* and *Bifidobacterium animalis* and an expansion of *Alistipes indistinctus*, *Clostridium hathewayi*, *Clostridium citroniae* and *Ruminococcus gauvreauii*, whilst no difference was observed in α diversity [74]. The study authors noted that the relative abundance of *B. animalis*, a species generally considered to confer health benefits, that they found in peritoneal dialysis patients was eightfold lower than what was reported in healthy controls [75]. Interestingly, randomised controlled trials have shown that sevelamer, a phosphate binder commonly prescribed to patients with CKD, is associated with reductions in serum AGEs in haemodialysis patients [76] and diabetic patients with stage 2–4 CKD [77]. In vitro work has illustrated that sevelamer binds AGE–BSA [78], indicating that it may lower serum AGEs by sequestering intestinal AGEs and limiting gastrointestinal absorption; however, whether sevelamer-bound AGEs are available for microbial metabolism in the colon is unknown. Further work is required in different human cohorts to assess the impact of high-AGE diets on the composition of the microbiome, and to link these changes to health outcomes.

### 7.3. Animal In Vivo Studies

Whilst there is a paucity of human studies, a number of studies have been undertaken using animals and assessed either the caecal or faecal microbiome following a high-AGE intervention. A contraction in *Lactobacilli* observed in humans following a high-AGE diet [73] and in vitro with glycated gluten [68] was confirmed by a rat study showing that diets supplemented with either the LMW fraction, the HMW fraction or total bread crust were associated with reductions in *Lactobacilli* compared with a control diet [79].

Animals (mice and catfish) that were fed thermally treated (steamed) fish had subsequent decreases in microbial α diversity compared with animals that were fed nonthermally treated fish [80]. Several animal studies have shown that heat-treated diets are associated with phylum-level changes in the composition of the gut microbiome, including a decrease in Bacteroidetes and an increase in Firmicutes [80,81], although these findings were not observed in rats consuming glycated fish protein [82]. The effects on *Bifidobacteria* have been inconsistent between studies, with a contraction of *Bifidobacteria* observed in rats following either a heat-treated diet [73] or supplementation with bread crust [79], although another study showed an expansion of *Bifidobacteria* following breast crust supplementation [83]. Oral administration of CML in mice with dextran sodium sulphate (DSS)-induced colitis was associated with a contraction in both Proteobacteria and *Enterobacteriaceae* [84]. Similar to the in vitro findings, there are discrepancies between studies, which limits our ability to draw broad conclusions. In general, high-AGE diets were associated with a contraction in Bacteroidetes, *Bifidobacteria*, *Lactobacilli* and α diversity; however, the findings of some studies contradict this. A summary of in vivo studies that showed the effects of AGEs on the microbiome is presented in Table 2.

## 8. Dietary AGEs and Microbial Production of Short-Chain Fatty Acids

### 8.1. In Vitro Studies

In addition to measuring changes in the composition of the microbiome following exposure to glycated food components, several in vitro studies have also assessed the effects of these diets on the production of short-chain fatty acids (SCFAs). When faecal samples from patients with ulcerative colitis were fermented with glycated BSA, there was no change in SCFA production relative to the control heated BSA [64], which was consistent with the results seen when faecal samples from healthy volunteers were incubated with glycated pea protein [67]. A study that utilised glycated gluten observed reductions in the three main SCFAs, acetate, propionate and butyrate, following fermentation [68], whilst fermentation with glycated fish protein was associated with an increase in acetate, but not propionate or butyrate [71]. There is considerable variation in the effects of high-AGE interventions on the production of SCFAs from human faecal samples in vitro.

### 8.2. In Vivo Studies

To date, no in vivo human studies that have utilised diets high in AGEs have reported concentrations of faecal SCFAs, thus the knowledge of the effects of high-AGE diets on these microbial metabolites comes from the results of animal studies. Glycated fish protein was associated with an increase in caecal and faecal butyrate concentrations [82], and heat-treated diets were associated with reductions in caecal SCFA concentrations [85,86], suggesting that high-AGE diet–induced alterations in the microbial composition led to a subsequent change in the production of microbial metabolites. Furthermore, high-AGE feeding in rats is associated with a decrease in mRNA and protein concentrations of occludin and zonula occludens-1, two tight junction proteins, indicating that high-AGE feeding is associated with increased intestinal permeability [85]. Whilst a thermally treated diet was associated with a reduction in SCFAs, a previous study using bread crust as a model of a high-MRP diet observed increased SCFA concentrations [79]. SCFA production was increased with supplementation with glycated fish protein, consistent with in vitro findings, or bread crust, but decreased when animals consumed baked chow.

## 9. Contradictory Findings

There remains some debate as to whether thermally treated diets are detrimental to the composition of the gut microbiome and, subsequently, health. This is due, in part, to the fact that not just AGEs are formed when foods are heat treated, but also other high-molecular-weight compounds, such as melanoidins, which can act as prebiotics in a bifidogenic fashion [87]. Results from in vitro studies have illustrated that a bifidogenic effect is observed with thermal treatment of bread crust [69], glycated gluten [68] and glycated pea protein [67]. However, in vivo it was observed that rats receiving bread crust–supplemented diets had a contraction of *Bifidobacteria* compared with control diets [79], and it was recently shown in an elegant experiment model utilising diets supplemented with either high-melanoidin bread crust, low-melanoidin bread crumb or a fibre-free melanoidin-free bread model that each diet had similarly bifidogenic effects compared with a control diet, indicating that the melanoidin content of bread is not responsible for the observed expansion of *Bifidobacteria* [83]. A previous report analysing the effect of bread crust melanoidins on the microbiome noted that there were large interindividual differences with regard to *Bifidobacteria*, but a consistent reduction in *Enterobacteria* across subjects [70].

Heat-treated diets have been found to be protective against inflammation and weight loss in a DSS-induced mouse model of colitis [88], which develops a dysbiotic microbiome [89]. Oral CML administration in the DSS-induced colitis mouse protected against weight loss and modulated the gut microbiome towards the control state, with a significant contraction of Proteobacteria [84], indicating that CML is in fact protective. Furthermore, the increased abundance of *Enterobacteriaceae* observed in DSS-induced colitis [89] was significantly reduced with CML administration [84]. Peritoneal dialysis patients often have decreased *B. animalis*, a beneficial bacterium frequently included in probiotic supplementation formulas, compared with healthy controls. Dietary AGE restriction in a population of peritoneal dialysis patients resulted in decreased *B. animalis*, suggesting that dietary AGEs may promote the growth of this beneficial bacteria [74]. The contradictory effects on the microbiome and health outcomes observed with heat-treated processed foods have been ascribed to the heterogeneity of compounds that are formed during thermal treatment [90] and requires further elucidation.

## 10. Conclusions

AGEs are formed exogenously in foods, particularly foods that are cooked at high temperatures for prolonged periods of time using dry heat. Dietary intake of AGEs has increased considerably with the adoption of the modern processed diet, and excessive AGE intake is associated with inflammation, oxidative stress and the progression of CKD, at least in animal models. The absorption of dietary AGEs is limited, with evidence indicating that the majority of protein-bound AGEs pass through the gastrointestinal tract to the colon, where they can act as substrates for the gut microbiota. Recent research has highlighted the detrimental role the microbiome can have in a number of pathological conditions, and there are conflicting reports on whether dietary AGEs negatively or positively influence the composition of the microbiome. Furthermore, there have been mixed reports on the effects of high-AGE diets on the production of SCFAs. Alteration of the microbiome by dietary AGEs is of particular interest, given the prevalence of these compounds in the modern processed diet.

## Figures and Tables

**Table 1:** Summary of in vitro studies.

Study	Stool Donors	High AGE Intervention	Control	*Bifidobacteria*	*Lactobacilli*	*Bacteroides*	*E. rectale*	*Escherichia/Shigella*	Other Microbiota	Total SCFAs	Acetic Acid	Propionic Acid	Butyric Acid
[64]	UC patients	Glycated BSA	Heated BSA	↓	-	↑	↓	-	↑ SRB, ↑ Clostridia	-	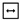	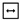	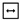
	Healthy donors	Glycated BSA	Heated BSA	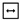	-	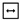	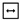	-	↑ SRB, 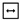 Clostridia	-	-	-	-
[68]	Healthy donors	Glycated gluten	Heated gluten	↑	↓	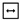	-	-		-	↓	↓	↓
[71]	Healthy donors	Glycated fish protein	Heated fish protein	-	-	↓	-	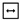	↓ Bact, ↑ Firm	↑	↑	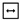	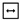
[70] ^1^	Healthy donors	Bread crust model	Prior to fermentation	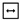	-	-	-	-	↓ Enterobacteria	-	-	-	-
[69]	Healthy donors	Bread	Prior to fermentation	↑	-	↓	-	-		-	-	-	-
[67]	Healthy donors	Glycated pea protein	Nonglycated pea protein	↑	↑	↑	-	-	↑ Enterobacteriacae	-	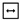	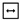	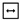

Bact, Bacteroidetes; BSA, bovine serum albumin; Firm, Firmicutes; SCFA, short-chain fatty acid, SRB, sulphate-reducing bacteria; UC, ulcerative colitis. ↑ = increased, ↓ = decreased, 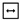 = no change, - = not reported. ^1^ Reported SCFAs as ratios rather than absolute values and was not included.

**Table 2:** Summary of in vivo studies.

Study	Model	High-AGE Intervention	Control	*Bifidobacteria*	*Lactobacilli*	*Bacteroides*	*C. Leptum*	*E. rectale*	*Escherichia/Shigella*	Other Microbiota	Total SCFAs	Acetic Acid	Propionic Acid	Butyric Acid
[73]	Healthy males	High MRP diet	Low MRP diet	↓	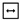	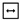	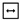	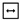	↓	↓ Enterobacteria	-	-	-	-
	Wistar rats, male	Heated AIN93G (150 °C for 90 m)	AIN93G	↓	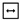	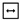	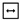	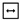	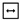	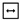 Enterobacteria	-	-	-	-
[79]	Wistar rats, male	Bread crust (10% diet)	AIN93G	↓	↓	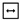	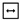	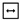	↑	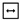 Enterobacteriacae	↑	↑	↑	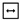
		HMW BC (10% diet)	AIN93G	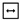	↓	↑	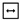	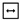	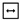	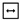 Enterobacteriacae	↑	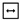	↑	↑
		LMW BC (10% diet)	AIN93G	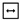	↓	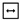	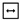	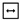	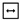	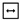 Enterobacteriacae	↑	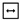	↑	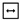
[80]	C57BL/6 mice, male	Steamed fish (15m)	Fish	-	-	↓	-	-	-	↓ α diversity, ↓ Bact, ↑ FB	-	-	-	-
	Catfish, male	Steamed fish (15m)	Fish	-	-	↑	-	-	-	↓ α diversity, ↓ Bact, ↓ Firm	-	-	-	-
[81]	ApoE^-/-^ mice, male	Heated high-fat diet (200 °C for 10 min)	High fat diet (40% E sat fat)	-	-	-	-	-	-	↓ Bact ↑ Firm, ↑ Clostridiales	-	-	-	-
[82]	SD rats, male	Glycated fish protein	Heated fish protein	-	-	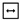	-	-	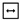	↓ Firm, ↑ Actinobacteria	↑	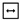	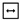	↑
[85]	SD rats, male	Heated AIN93G (125 °C for 180 min)	AIN93G	-	-	↑	-	-	-	↓ α diversity, ↓ Bact, ↑ Proteobacteria	-	↓	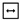	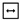
[86]	C57BL/6 mice, male	Heated AIN93G (175 °C for 45 min)	AIN93G	-	-	↓	-	-	-	↓ Firm, ↑ Actinobacteria, ↓ Clostridia	-	↓	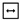	↓
[83]	SD rats, male	Bread crust (13% diet)	A04	↑	-	-	-	-	-	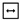 Bact, 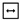 Firm	-	-	-	-
[84]	Balb/c mice, male, DSS-induced colitis	CML (1.6 mg/kg/d)	Saline	-	-	-	-	-	-	↓ Proteobacteria, ↓ Enterobacteriacae	-	-	-	-
	Balb/c mice, male, healthy	CML (1.6 mg/kg/d)	Saline	-	-	-	-	-	-	↓ Lachnospiraceae, ↑ Desulfovibrionaceae	-	-	-	-
[74]	Peritoneal dialysis patients	Usual diet (high AGE)	Dietary AGE restriction	-	-	-	-	-	-	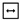 α diversity, ↑ *Prevotella copri*, ↑ *B. animalis*	-	-	-	-

Bact, Bacteroidetes; FB, Firmicutes; SCFA, short-chain fatty acid; SD, Sprague-Dawley; MRP, Maillard reaction product; CML carboxymethyl–lysine. ↑ = increased, ↓ = decreased, 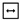 = no change, - = not reported.

## References

[1] Schalkwijk C.G., Miyata T. (2012). Early- and advanced non-enzymatic glycation in diabetic vascular complications: The search for therapeutics. Amino Acids.

[2] Poulsen M.W., Hedegaard R.V., Andersen J.M., de Courten B., Bügel S., Nielsen J., Skibsted L.H., Dragsted L.O. (2013). Advanced glycation endproducts in food and their effects on health. Food Chem. Toxicol..

[3] Wrobel K., Wrobel K., Ortiz S.J., Escobosa A.R.C., Uribarri J. (2018). What are ages, their chemical structure, and how can they be measured?. Dietary Ages and Their Role in Health and Disease.

[4] Guilbaud A., Niquet-Leridon C., Boulanger E., Tessier F.J. (2016). How can diet affect the accumulation of advanced glycation end-products in the human body?. Foods.

[5] Rabbani N., Thornalley P.J. (2018). Advanced glycation end products in the pathogenesis of chronic kidney disease. Kidney Int..

[6] Nowotny K., Jung T., Grune T., Höhn A. (2014). Accumulation of modified proteins and aggregate formation in aging. Exp. Gerontol..

[7] Rhee S.Y., Kim Y.S. (2018). The role of advanced glycation end products in diabetic vascular complications. Diabetes Metab. J..

[8] Aragno M., Mastrocola R. (2017). Dietary sugars and endogenous formation of advanced glycation endproducts: Emerging mechanisms of disease. Nutrients.

[9] Rabbani N., Thornalley P.J. (2015). Dicarbonyl stress in cell and tissue dysfunction contributing to ageing and disease. Biochem. Biophys. Res. Commun..

[10] Cerami C., Founds H., Nicholl I., Mitsuhashi T., Giordano D., Vanpatten S., Lee A., Al-Abed Y., Vlassara H., Bucala R. (1997). Tobacco smoke is a source of toxic reactive glycation products. Proc. Natl. Acad. Sci. USA.

[11] Berg T.J., Snorgaard O., Faber J., Torjesen P.A., Hildebrandt P., Mehlsen J., Hanssen K.F. (1999). Serum levels of advanced glycation end products are associated with left ventricular diastolic function in patients with type 1 diabetes. Diabetes Care.

[12] Nicholl I.D., Stitt A.W., Moore J.E., Ritchie A.J., Archer D.B., Bucala R. (1998). Increased levels of advanced glycation endproducts in the lenses and blood vessels of cigarette smokers. Mol. Med. (Camb. Mass.).

[13] Scheijen J.L.J.M., Clevers E., Engelen L., Dagnelie P.C., Brouns F., Stehouwer C.D.A., Schalkwijk C.G. (2016). Analysis of advanced glycation endproducts in selected food items by ultra-performance liquid chromatography tandem mass spectrometry: Presentation of a dietary age database. Food Chem..

[14] Hull G.L.J., Woodside J.V., Ames J.M., Cuskelly G.J. (2012). Nε-(carboxymethyl)lysine content of foods commonly consumed in a western style diet. Food Chem..

[15] Helou C., Gadonna-Widehem P., Robert N., Branlard G., Thebault J., Librere S., Jacquot S., Mardon J., Piquet-Pissaloux A., Chapron S. (2016). The impact of raw materials and baking conditions on maillard reaction products, thiamine, folate, phytic acid and minerals in white bread. Food Funct..

[16] Nowotny K., Schröter D., Schreiner M., Grune T. (2018). Dietary advanced glycation end products and their relevance for human health. Ageing Res. Rev..

[17] Uribarri J., Woodruff S., Goodman S., Cai W., Chen X., Pyzik R., Yong A., Striker G.E., Vlassara H. (2010). Advanced glycation end products in foods and a practical guide to their reduction in the diet. J. Am. Diet. Assoc..

[18] Delgado-Andrade C. (2016). Carboxymethyl-lysine: Thirty years of investigation in the field of age formation. Food Funct..

[19] Henle T. (2005). Protein-bound advanced glycation endproducts (ages) as bioactive amino acid derivatives in foods. Amino Acids.

[20] Henle T., Miyata T. (2003). Advanced glycation end products in uremia. Adv. Ren. Replace. Ther..

[21] Scheijen J.L.J.M., Hanssen N.M.J., van Greevenbroek M.M., Van der Kallen C.J., Feskens E.J.M., Stehouwer C.D.A., Schalkwijk C.G. (2018). Dietary intake of advanced glycation endproducts is associated with higher levels of advanced glycation endproducts in plasma and urine: The codam study. Clin. Nutr..

[22] Kellow N.J., Coughlan M.T. (2015). Effect of diet-derived advanced glycation end products on inflammation. Nutr. Rev..

[23] Vlassara H., Cai W., Crandall J., Goldberg T., Oberstein R., Dardaine V., Peppa M., Rayfield E.J. (2002). Inflammatory mediators are induced by dietary glycotoxins, a major risk factor for diabetic angiopathy. Proc. Natl. Acad. Sci. USA.

[24] Semba R.D., Gebauer S.K., Baer D.J., Sun K., Turner R., Silber H.A., Talegawkar S., Ferrucci L., Novotny J.A. (2014). Dietary intake of advanced glycation end products did not affect endothelial function and inflammation in healthy adults in a randomized controlled trial. J. Nutr..

[25] Harcourt B.E., Sourris K.C., Coughlan M.T., Walker K.Z., Dougherty S.L., Andrikopoulos S., Morley A.L., Thallas-Bonke V., Chand V., Penfold S.A. (2011). Targeted reduction of advanced glycation improves renal function in obesity. Kidney Int..

[26] Baye E., Kiriakova V., Uribarri J., Moran L.J., de Courten B. (2017). Consumption of diets with low advanced glycation end products improves cardiometabolic parameters: Meta-analysis of randomised controlled trials. Sci. Rep..

[27] Uribarri J., Stirban A., Sander D., Cai W., Negrean M., Buenting C.E., Koschinsky T., Vlassara H. (2007). Single oral challenge by advanced glycation end products acutely impairs endothelial function in diabetic and nondiabetic subjects. Diabetes Care.

[28] Patel R., Baker S.S., Liu W., Desai S., Alkhouri R., Kozielski R., Mastrandrea L., Sarfraz A., Cai W., Vlassara H. (2012). Effect of dietary advanced glycation end products on mouse liver. PLoS ONE.

[29] Leung C., Herath C.B., Jia Z., Goodwin M., Mak K.Y., Watt M.J., Forbes J.M., Angus P.W. (2014). Dietary glycotoxins exacerbate progression of experimental fatty liver disease. J. Hepatol..

[30] Abate G., Marziano M., Rungratanawanich W., Memo M., Uberti D. (2017). Nutrition and age-ing: Focusing on alzheimer’s disease. Oxidative Med. Cell. Longev..

[31] Šebeková K., Hofmann T., Boor P., Šebeková K., Ulicná O.G., Erbersdobler H.F., Baynes J.W., Thorpe S.R., Heidland A., Somoza V. (2005). Renal effects of oral maillard reaction product load in the form of bread crusts in healthy and subtotally nephrectomized rats. Ann. N. Y. Acad. Sci..

[32] Šebeková K., Faist V., Hofmann T., Schinzel R., Heidland A. (2003). Effects of a diet rich in advanced glycation end products in the rat remnant kidney model. Am. J. Kidney Dis. Off. J. Natl. Kidney Found..

[33] Zheng F., He C., Cai W., Hattori M., Steffes M., Vlassara H. (2002). Prevention of diabetic nephropathy in mice by a diet low in glycoxidation products. Diabetes/Metab. Res. Rev..

[34] Peppa M., Uribarri J., Cai W., Lu M., Vlassara H. (2004). Glycoxidation and inflammation in renal failure patients. Am. J. Kidney Dis..

[35] Vlassara H., Cai W., Goodman S., Pyzik R., Yong A., Chen X., Zhu L., Neade T., Beeri M., Silverman J.M. (2009). Protection against loss of innate defenses in adulthood by low advanced glycation end products (age) intake: Role of the antiinflammatory age receptor-1. J. Clin. Endocrinol. Metab..

[36] Singh R., Barden A., Mori T., Beilin L. (2001). Advanced glycation end-products: A review. Diabetologia.

[37] Shimizu F., Sano Y., Tominaga O., Maeda T., Abe M.A., Kanda T. (2013). Advanced glycation end-products disrupt the blood-brain barrier by stimulating the release of transforming growth factor-beta by pericytes and vascular endothelial growth factor and matrix metalloproteinase-2 by endothelial cells in vitro. Neurobiol. Aging.

[38] Haucke E., Navarrete-Santos A., Simm A., Silber R.-E., Hofmann B. (2014). Glycation of extracellular matrix proteins impairs migration of immune cells. Wound Repair Regen..

[39] Akhter F., Salman Khan M., Shahab U., Moinuddin, Ahmad S. (2013). Bio-physical characterization of ribose induced glycation: A mechanistic study on DNA perturbations. Int. J. Biol. Macromol..

[40] Stopper H., Schupp N., Klassen A., Sebekova K., Heidland A. (2005). Genomic damage in chronic renal failure—Potential therapeutic interventions. J. Ren. Nutr..

[41] Yim M.B., Yim H.-S., Lee C., Kang S.-O., Chock P.B. (2001). Protein glycation. Ann. N. Y. Acad. Sci..

[42] Ott C., Jacobs K., Haucke E., Navarrete Santos A., Grune T., Simm A. (2014). Role of advanced glycation end products in cellular signaling. Redox Biol..

[43] Nedić O., Rattan S.I.S., Grune T., Trougakos I.P. (2013). Molecular effects of advanced glycation end products on cell signalling pathways, ageing and pathophysiology. Free Radic. Res..

[44] Liu Y., Liang C., Liu X., Liao B., Pan X., Ren Y., Fan M., Li M., He Z., Wu J. (2010). Ages increased migration and inflammatory responses of adventitial fibroblasts via rage, mapk and nf-κb pathways. Atherosclerosis.

[45] Coughlan M.T., Thorburn D.R., Penfold S.A., Laskowski A., Harcourt B.E., Sourris K.C., Tan A.L.Y., Fukami K., Thallas-Bonke V., Nawroth P.P. (2009). Rage-induced cytosolic ros promote mitochondrial superoxide generation in diabetes. J. Am. Soc. Nephrol..

[46] Riehl A., Németh J., Angel P., Hess J. (2009). The receptor rage: Bridging inflammation and cancer. Cell Commun. Signal..

[47] Gugliucci A., Menini T., Jordi C. (2014). The axis age-rage-soluble rage and oxidative stress in chronic kidney disease. Oxidative Stress and Inflammation in Non-Communicable Diseases—Molecular Mechanisms and Perspectives in Therapeutics.

[48] Koschinsky T., He C.-J., Mitsuhashi T., Bucala R., Liu C., Buenting C., Heitmann K., Vlassara H. (1997). Orally absorbed reactive glycation products (glycotoxins): An environmental risk factor in diabetic nephropathy. Proc. Natl. Acad. Sci. USA.

[49] He C., Sabol J., Mitsuhashi T., Vlassara H. (1999). Inhibition of reactive products by aminoguanidine facilitates renal clearance and reduces tissue sequestration. Diabetes.

[50] Grunwald S., Krause R., Bruch M., Henle T., Brandsch M. (2006). Transepithelial flux of early and advanced glycation compounds across caco-2 cell monolayers and their interaction with intestinal amino acid and peptide transport systems. Br. J. Nutr..

[51] Hellwig M., Geissler S., Peto A., Knütter I., Brandsch M., Henle T. (2009). Transport of free and peptide-bound pyrraline at intestinal and renal epithelial cells. J. Agric. Food Chem..

[52] Geissler S., Hellwig M., Zwarg M., Markwardt F., Henle T., Brandsch M. (2010). Transport of the advanced glycation end products alanylpyrraline and pyrralylalanine by the human proton-coupled peptide transporter hpept1. J. Agric. Food Chem..

[53] Hellwig M., Geissler S., Matthes R., Peto A., Silow C., Brandsch M., Henle T. (2011). Transport of free and peptide-bound glycated amino acids: Synthesis, transepithelial flux at caco-2 cell monolayers, and interaction with apical membrane transport proteins. ChemBioChem.

[54] Forster A., Kuhne Y., Henle T. (2005). Studies on absorption and elimination of dietary maillard reaction products. Ann. N. Y. Acad. Sci..

[55] Roncero-Ramos I., Delgado-Andrade C., Tessier F.J., Niquet-Léridon C., Strauch C., Monnier V.M., Navarro M.P. (2013). Metabolic transit of nε-carboxymethyl-lysine after consumption of ages from bread crust. Food Funct..

[56] Hellwig M., Matthes R., Peto A., Lobner J., Henle T. (2014). N-epsilon-fructosyllysine and n-epsilon-carboxymethyllysine, but not lysinoalanine, are available for absorption after simulated gastrointestinal digestion. Amino Acids.

[57] Faist V., Erbersdobler H.F. (2001). Metabolic transit and in vivo effects of melanoidins and precursor compounds deriving from the maillard reaction. Ann. Nutr. Metab..

[58] Tessier F.J., Niquet-Léridon C., Jacolot P., Jouquand C., Genin M., Schmidt A.-M., Grossin N., Boulanger E. (2016). Quantitative assessment of organ distribution of dietary protein-bound 13c-labeled nε-carboxymethyllysine after a chronic oral exposure in mice. Mol. Nutr. Food Res..

[59] Delgado-Andrade C., Tessier F.J., Niquet-Leridon C., Seiquer I., Pilar Navarro M. (2012). Study of the urinary and faecal excretion of nε-carboxymethyllysine in young human volunteers. Amino Acids.

[60] Alamir I., Niquet-leridon C., Jacolot P., Rodriguez C., Orosco M., Anton P.M., Tessier F.J. (2013). Digestibility of extruded proteins and metabolic transit of nε-carboxymethyllysine in rats. Amino Acids.

[61] Garay-Sevilla M.E., Gómez-Ojeda A., Luevano-Contreras C., Uribarri J. (2018). Dietary ages and diabetic complications. Dietary Ages and Their Role in Health and Disease.

[62] Lanciotti R., Anese M., Sinigaglia M., Severini C., Massini R. (1999). Effects of heated glucose-fructose-glutamic acid solutions on the growth ofbacillus stearothermophilus. LWT Food Sci. Technol..

[63] Monnier V.M. (2005). Bacterial enzymes that can deglycate glucose- and fructose-modified lysine. Biochem. J..

[64] Mills D.J.S., Tuohy K.M., Booth J., Buck M., Crabbe M.J.C., Gibson G.R., Ames J.M. (2008). Dietary glycated protein modulates the colonic microbiota towards a more detrimental composition in ulcerative colitis patients and non-ulcerative colitis subjects. J. Appl. Microbiol..

[65] Barada K.A., Kafrouni M.I., Khoury C.I., Saade N.E., Mourad F.H., Szabo S.S., Nassar C.F. (2001). Experimental colitis decreases rat jejunal amino acid absorption: Role of capsaicin sensitive primary afferents. Life Sci..

[66] Barada K., Mourad F.H., Noutsi B., Saadé N.E. (2015). Electrocautery-induced localized colonic injury elicits increased levels of pro-inflammatory cytokines in small bowel and decreases jejunal alanine absorption. Cytokine.

[67] Świątecka D., Narbad A., Ridgway K.P., Kostyra H. (2011). The study on the impact of glycated pea proteins on human intestinal bacteria. Int. J. Food Microbiol..

[68] Dell’Aquila C., Ames J.M., Gibson G.R., Wynne A.G. (2003). Fermentation of heated gluten systems by gut microflora. Eur. Food Res. Technol..

[69] Borrelli R.C., Fogliano V. (2005). Bread crust melanoidins as potential prebiotic ingredients. Mol. Nutr. Food Res..

[70] Helou C., Denis S., Spatz M., Marier D., Rame V., Alric M., Tessier F.J., Gadonna-Widehem P. (2015). Insights into bread melanoidins: Fate in the upper digestive tract and impact on the gut microbiota using in vitro systems. Food Funct..

[71] Yang Y., Wu H., Dong S., Jin W., Han K., Ren Y., Zeng M. (2018). Glycation of fish protein impacts its fermentation metabolites and gut microbiota during in vitro human colonic fermentation. Food Res. Int..

[72] Hellwig M., Bunzel D., Huch M., Franz C.M.A.P., Kulling S.E., Henle T. (2015). Stability of individual maillard reaction products in the presence of the human colonic microbiota. J. Agric. Food Chem..

[73] Seiquer I., Rubio L.A., Peinado M.J., Delgado-Andrade C., Navarro M.P. (2014). Maillard reaction products modulate gut microbiota composition in adolescents. Mol. Nutr. Food Res..

[74] Yacoub R., Nugent M., Cai W., Nadkarni G.N., Chaves L.D., Abyad S., Honan A.M., Thomas S.A., Zheng W., Valiyaparambil S.A. (2017). Advanced glycation end products dietary restriction effects on bacterial gut microbiota in peritoneal dialysis patients; a randomized open label controlled trial. PLoS ONE.

[75] Turroni F., Foroni E., Pizzetti P., Giubellini V., Ribbera A., Merusi P., Cagnasso P., Bizzarri B., de’Angelis G.L., Shanahan F. (2009). Exploring the diversity of the bifidobacterial population in the human intestinal tract. Appl. Environ. Microbiol..

[76] Kakuta T., Tanaka R., Hyodo T., Suzuki H., Kanai G., Nagaoka M., Takahashi H., Hirawa N., Oogushi Y., Miyata T. (2011). Effect of sevelamer and calcium-based phosphate binders on coronary artery calcification and accumulation of circulating advanced glycation end products in hemodialysis patients. Am. J. Kidney Dis..

[77] Yubero-Serrano E.M., Woodward M., Poretsky L., Vlassara H., Striker G.E. (2015). Effects of sevelamer carbonate on advanced glycation end products and antioxidant/pro-oxidant status in patients with diabetic kidney disease. Clin. J. Am. Soc. Nephrol..

[78] Vlassara H., Uribarri J., Cai W., Goodman S., Pyzik R., Post J., Grosjean F., Woodward M., Striker G.E. (2012). Effects of sevelamer on hba1c, inflammation, and advanced glycation end products in diabetic kidney disease. Clin. J. Am. Soc. Nephrol..

[79] Delgado-Andrade C., Pastoriza de la Cueva S., Peinado M.J., Rufián-Henares J.Á., Navarro M.P., Rubio L.A. (2017). Modifications in bacterial groups and short chain fatty acid production in the gut of healthy adult rats after long-term consumption of dietary maillard reaction products. Food Res. Int..

[80] Zhang Z., Li D. (2018). Thermal processing of food reduces gut microbiota diversity of the host and triggers adaptation of the microbiota: Evidence from two vertebrates. Microbiome.

[81] Marungruang N., Fåk F., Tareke E. (2016). Heat-treated high-fat diet modifies gut microbiota and metabolic markers in apoe−/− mice. Nutr. Metab. Lond..

[82] Han K., Jin W., Mao Z., Dong S., Zhang Q., Yang Y., Chen B., Wu H., Zeng M. (2018). Microbiome and butyrate production are altered in the gut of rats fed a glycated fish protein diet. J. Funct. Foods.

[83] Helou C., Anton P.M., Niquet-Léridon C., Spatz M., Tessier F.J., Gadonna-Widehem P. (2017). Fecal excretion of maillard reaction products and the gut microbiota composition of rats fed with bread crust or bread crumb. Food Funct..

[84] Aljahdali N., Gadonna-Widehem P., Delayre-Orthez C., Marier D., Garnier B., Carbonero F., Anton P.M. (2017). Repeated oral exposure to nε-carboxymethyllysine, a maillard reaction product, alleviates gut microbiota dysbiosis in colitic mice. Dig. Dis. Sci..

[85] Qu W., Yuan X., Zhao J., Zhang Y., Hu J., Wang J., Li J. (2017). Dietary advanced glycation end products modify gut microbial composition and partially increase colon permeability in rats. Mol. Nutr. Food Res..

[86] Qu W., Nie C., Zhao J., Ou X., Zhang Y., Yang S., Bai X., Wang Y., Wang J., Li J. (2018). Microbiome-metabolomics analysis of the impacts of long-term dietary advanced glycation end products consumption on the c57bl/6 mouse fecal microbiota and metabolite perturbation. J. Agric. Food Chem..

[87] Tagliazucchi D., Bellesia A. (2015). The gastro-intestinal tract as the major site of biological action of dietary melanoidins. Amino Acids.

[88] Anton P.M., Craus A., Niquet-Léridon C., Tessier F.J. (2012). Highly heated food rich in maillard reaction products limit an experimental colitis in mice. Food Funct..

[89] Munyaka P.M., Rabbi M.F., Khafipour E., Ghia J.-E. (2016). Acute dextran sulfate sodium (dss)-induced colitis promotes gut microbial dysbiosis in mice. J. Basic Microbiol..

[90] Zinöcker M.K., Lindseth I.A. (2018). The western diet–microbiome-host interaction and its role in metabolic disease. Nutrients.

